# Testing the excitation/inhibition imbalance hypothesis in a mouse model of the autism spectrum disorder: in vivo neurospectroscopy and molecular evidence for regional phenotypes

**DOI:** 10.1186/s13229-017-0166-4

**Published:** 2017-09-19

**Authors:** Joana Gonçalves, Inês R. Violante, José Sereno, Ricardo A. Leitão, Ying Cai, Antero Abrunhosa, Ana Paula Silva, Alcino J. Silva, Miguel Castelo-Branco

**Affiliations:** 1 CiBIT, Institute for Nuclear Sciences Applied to Health (ICNAS), University of Coimbra, Coimbra, Portugal; 2 Institute for Biomedical Imaging and Life Sciences (IBILI), Faculty of Medicine, University of Coimbra, Coimbra, Portugal; 3 Center for Neuroscience and Cell Biology-Institute for Biomedical Imaging and Life Sciences (CNC.IBILI) Research Unit, University of Coimbra, Coimbra, Portugal; 4 School of Psychology, Faculty of Health and Medical Sciences, University of Surrey, Guildford, UK; 5 Laboratory of Pharmacology and Experimental Therapeutics, Faculty of Medicine, University of Coimbra, Coimbra, Portugal; 6 Department of Neurobiology, Integrative Center for Learning and Memory, Brain Research Institute, University of California Los Angeles, Los Angeles, CA USA

**Keywords:** Autism spectrum disorders, Neurofibromatosis type 1, Excitation/inhibition imbalance, GABA(A) receptor, Magnetic resonance spectroscopy

## Abstract

**Background:**

Excitation/inhibition (E/I) imbalance remains a widely discussed hypothesis in autism spectrum disorders (ASD). The presence of such an imbalance may potentially define a therapeutic target for the treatment of cognitive disabilities related to this pathology. Consequently, the study of monogenic disorders related to autism, such as neurofibromatosis type 1 (NF1), represents a promising approach to isolate mechanisms underlying ASD-related cognitive disabilities. However, the NF1 mouse model showed increased γ-aminobutyric acid (GABA) neurotransmission, whereas the human disease showed reduced cortical GABA levels. It is therefore important to clarify whether the E/I imbalance hypothesis holds true. We hypothesize that E/I may depend on distinct pre- and postsynaptic push-pull mechanisms that might be are region-dependent.

**Methods:**

In current study, we assessed two critical components of E/I regulation: the concentration of neurotransmitters and levels of GABA(A) receptors. Measurements were performed across the hippocampi, striatum, and prefrontal cortices by combined in vivo magnetic resonance spectroscopy (MRS) and molecular approaches in this ASD-related animal model, the *Nf1* ^+/−^ mouse.

**Results:**

Cortical and striatal GABA/glutamate ratios were increased. At the postsynaptic level, very high receptor GABA(A) receptor expression was found in hippocampus, disproportionately to the small reduction in GABA levels. Gabaergic tone (either by receptor levels change or GABA/glutamate ratios) seemed therefore to be enhanced in all regions, although by a different mechanism.

**Conclusions:**

Our data provides support for the hypothesis of E/I imbalance in NF1 while showing that pre- and postsynaptic changes are region-specific. All these findings are consistent with our previous physiological evidence of increased inhibitory tone. Such heterogeneity suggests that therapeutic approaches to address neurochemical imbalance in ASD may need to focus on targets where convergent physiological mechanisms can be found.

## Background

The E/I imbalance hypothesis, conceptualized as a disequilibrium between the glutamatergic and γ-aminobutyric acidergic (GABAergic) inputs, has been postulated to underlie brain dysfunction across neurodevelopmental and neuropsychiatric disorders, including autism spectrum disorder (ASD) and schizophrenia (SCZ) [1, 2]. According to this framework, changes in the balance between these neurotransmitters impact excitation/inhibition ratios altering brain activity patterns and resulting in behavioral and cognitive impairments. In the wide range of autism spectrum disorders (ASD), neurofibromatosis type 1 (NF1) is a relevant model to study this hypothesis. This common monogenic developmental disorder, characterized by altered skin pigmentation and increased tumor predisposition, is associated with macrocephaly and T2-weighted hyperintensities [3]. Consequently, children with NF1 commonly show cognitive deficits in visuospatial, motor, language, and memory domains leading to significant delays in neurodevelopmental milestones and often reduced intelligent quotient (IQ) [4, 5]. The similarities between the cognitive phenotypes of NF1 and idiopathic ASD make this single-gene disorder a relevant model of syndromic autism [6, 7].

NF1 is caused by a single mutation of the *Nf1* gene that encodes for neurofibromin, a negative regulator of the RAS pathway. This protein is expressed in both neurons and glia and is required for the regulation of cellular growth and differentiation [3]. Previous studies employing a mouse model of NF1 demonstrated that γ-aminobutyric acid (GABA) inhibition was enhanced in the hippocampus due to excessive RAS activity caused by mutations in the *Nf1* gene [8–12]. These alterations in GABA neurotransmission were associated with learning and memory deficits [8–12]. In humans, we have found that excitation/inhibition balance is altered in the visual and medial frontal cortex of patients with NF1, as a consequence of reduced GABA concentration [13, 14]. More recently, we observed a decreased binding of GABA(A) receptors (GABA(A)R) in patients in the parieto-occipital cortex, midbrain, and thalamus suggesting neurodevelopmental synaptopathy both at the pre- and postsynaptic level [15, 16]. However, levels of GABA and GABA(A)R have never been assessed in NF1 mutant mice, which is important to link the preclinical model to clinical observations and to the development of therapies targeting GABAergic pathways. For example, picrotoxin, a GABA(A)R inhibitor, has been shown to reverse learning deficits in the NF1 mouse model [9, 10]. Overall, although both animal and human studies demonstrate an excitation/inhibition imbalance in NF1, the nature of such disequilibrium remains to be understood, both across species and brain regions.

The present aimed to directly test the E/I imbalance hypothesis in an animal model of NF1. We focused on two critical elements contributing to the E/I balance: (1) the concentration of GABA, the main inhibitory neurotransmitter, and glutamate, the main excitatory neurotransmitter; (2) the postsynaptic levels of the GABA(A) receptor. In an effort to close the gap between preclinical models and human disease, the concentration of neurotransmitters was assessed using magnetic resonance spectroscopy (MRS), the same technique as available to measure GABA levels in vivo in humans. This was then combined with cellular/molecular approaches with the aim of providing a comprehensive view of synaptic function, by investigating GABA(A) receptor levels. We focused on three brain regions, the hippocampus, prefrontal cortex, and striatum, as these regions have previously been shown by us and others to be affected in the NF1 model, leading to increased inhibitory activity, and in patients [8–10, 14, 16].

Our findings suggest region-specific imbalances which involve GABA pools and receptor levels. The fact that E/I imbalance is of a distinct nature at different brain regions, although all leading to increased inhibitory drive, suggests that therapeutic approaches to restore E/I balance need to address this regional heterogeneity, possibly by choosing upstream targets.

## Methods

### Animals

Fourteen *Nf1* ^*+/−*^ mice were used in the experiments; these mice were obtained from animals backcrossed to Taconic C57Bl/6 mice at least 10 times and bred once with 129T2/SvEmsJ before experiments. This breeding procedure was put in place to keep the genetic background of the *Nf1* ^+/−^ mice constant across experiments and studies [17, 18]. Seven- to eight-month-old male and female mice were used. All experiments used littermates as controls (*n* = 13) and were carried and analyzed with the experimenters blinded to genotype and treatment. Animals were group housed (2–4) on a 12-h light/dark cycle in vivarium at UCLA, CAU, and CNC.IBILI. The experiments were carried out in accordance with the European Union Council Directive (2010/63/EU), the National Regulations, and the Internal Review Board of the University of Coimbra. Experimental protocols at the University of California were approved by the Chancellor’s Animal Research Committee of the University of California, Los Angeles, in accordance with NIH guidelines. All included animals were healthy (discomfort score 0), and all efforts were made to minimize the number of animals used and their suffering.

### ^1^H-MRS

Acquisitions were performed on a 9.4T MR pre-clinical scanner (Bruker Biospec, Billerica MA) equipped with a standard Bruker crosscoil setup using a volume coil for excitation (86/112 mm of inner/outer diameter) and quadrature surface coil for detection (Bruker Biospin, Ettlingen, Germany). Mice were anesthetized by inhalation of 1.5% isoflurane with 100% O_2_ and placed on temperature-controlled animal beds. A tooth bar and head restrain were used to reduce motion artifacts. Physiological parameters were continuously monitored and body temperature was maintained at 37 °C using a mouse heating cover (Bruker Biospin, Ettlingen, Germany). To assure accurate voxel positioning, we first obtained T2-weighted images in axial, coronal, and sagittal planes using a rapid multi-slice acquisition refocusing echoes (RARE) sequence directions with the following parameters: repetition time (TR) = 2500 ms, echo time (TE) = 33 ms, matrix size 256 × 256, field of view 20 × 20 mm^2^, 22 slices, slice thickness = 0.5 mm, RARE factor = 8, and 1 average. First- and second-order shims were adjusted using the fieldmap-based MAPSHIM routine, leading to water linewidths between 14 and 20 Hz. MRS voxels were positioned in three locations: striatum (1.8 × 2.0 × 2.2 mm), hippocampus (1.9 × 1.3 × 1.9 mm), and pre-frontal cortex (2.0 × 1.2 × 2.0 mm). Spectra were acquired using a point-resolved spectroscopy (PRESS) sequence with outer volume suppression (OVS) and VAPOR water suppression [19, 20]. The following parameters were used: TR = 2500 ms, TE = 16,225 ms, number of averages = 720, 3 flip angles = 90°, 142°, 142°, bandwidth = 5000 Hz, number of acquired points = 2048 yielding a spectral resolution of 1.22 Hz/pt. Total acquisition time was 30 min. Before each spectrum, we acquired an unsuppressed water spectrum at the same voxel location (TE = 16,225 ms, TR = 2500 ms, 16 averages, scanning time = 40 s). Data analysis of ^1^H-MRS spectra was performed using linear combination modeling LCModel (Stephen Provencher Inc., Toronto, Canada) [21]. Metabolite quantification was performed applying the internal water reference method. Concentrations in millmole units were calculated for metabolites, and results are presented in institutional units (i.u.). Only metabolites with Cramér–Rao bounds < 20% were considered for statistical analysis.

### Western blot

Mice were sacrificed following the ^1^H-MRS procedure. Brains from half of the animals (7 *Nf1* ^+/−^ and 7 WT mice) were removed, and the hippocampi, prefrontal cortex, and striata were dissected on ice, to isolate the synaptossomal fraction using Syn-PER Synaptic Protein Extraction Reagent (Thermo Scientific, Pierce Biotechnology, Rockford, USA) according to the manufacturer’s instructions. The protein fraction was then quantified using the BCA method and stored at − 80 °C until further use. The proteins were separated on SDS-polyacrylamide gel electrophoresis and transferred onto polyvinylidine difluoride membrane (PVDF; Milipore, Madrid, Spain). After blocked, membranes were incubated with anti-GABA(A)R α1 (1:1000; ref. AGA-001 from Alomone Labs, Jerusalem, Israel), washed and then incubated with alkaline phosphatase-conjugated secondary antibodies (1:20,000; ref. RPN4301 from Amersham, GE Healthcare Life Science, USA) and visualized using ECF reagent (Amersham) on Typhoon FLA 9000 (GE Healthcare Bio-Science AB, Uppsala, Sweden). Immunoblots were reprobed with β-actin antibody (1:10,000; ref. A5441 from Sigma-Aldrich) to ensure equal sample loading, and densitometric analyses were performed using the NIH ImageJ 1.44 analysis software.

### Immunohistochemistry

Following imaging analysis, the other half of animals (7 *Nf1* ^+/−^ and 6 WT mice) were sacrificed and their brains were removed, frozen on dry ice, and stored at − 80 °C. Coronal sections (14 μm) were cut on a cryostat (Thermo Shandon Inc.) from the posterior to anterior brain. Before starting the immunohistochemistry procedure, the brain slices were fixed in 4% paraformaldehyde (PFA). Then, immunolabeling was performed for the GABA(A)α1 receptor subunit. Briefly, brain sections were rinsed, blocked, and incubated with anti-GABA(A)α1 (1:2000; ref. AGA-001 from Alomone Labs). Brain slices were then washed and incubated with Alexa Fluor 488 anti-rabbit secondary antibody (1:200; ref. ab150077 from Invitrogen, Inchinnan, Business Park, UK). Sections were again washed and stained with Hoechst 33342 (5 μg/mL; ref. 94403 from Sigma-Aldrich), mounted in Dako fluorescence medium (Dako North America, Carpinteria, USA), and images were recorded using LSM 710 Meta Confocal microscope (Carl Zeiss, Oberkochen, Germany). The quantification of fluorescence intensities of GABA(A)α1 receptor labeling was performed using the NIH Fiji ImageJ 2.0.0 analysis software.

### Statistics

Data are expressed as mean ± SEM and are statistically different at *p* < 0.05. Statistical analysis was performed using the Mann-Whitney test in GraphPad Prism 6.0 (GraphPad software, Inc., San Diego, CA, USA). Additionally, statistical comparisons concerning metabolite concentrations for each brain region were controlled for using the Benjamini-Hochberg false discovery rate procedure.

## Results

**Fig. 1:**
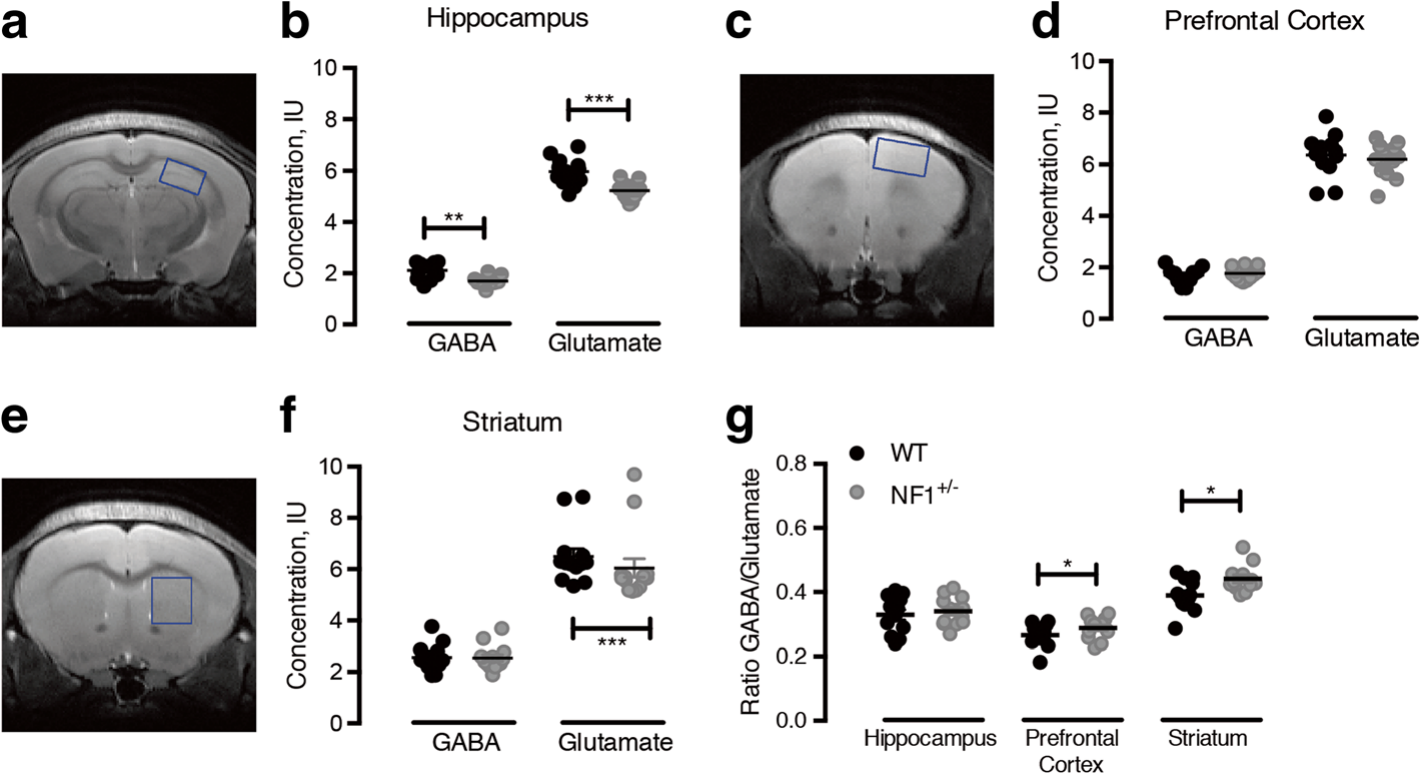
*Nf1* ^*+/−*^ mutation induces imbalance of excitatory/inhibitory systems. Localization of the magnetic resonance spectroscopy (MRS) voxel (blue square) in the hippocampus (HIP; **a**), prefrontal cortex (PFC; **c**), and striatum (STR; **e**) of representative *Nf1* ^*+/−*^ mice. GABA and glutamate levels in hippocampus (**b**), prefrontal cortex (**d**), and striatum (**f**) of wild-type (black circle) and *Nf1* ^*+/−*^ (gray circle) mice. MRS measurements revealed that hippocampal GABA and glutamate concentration are decreased (**b**) as well as striatal levels of glutamate (**f**). However, no changes are found in *Nf1* ^*+/−*^ prefrontal cortex (**d**). The analyses indicated an upregulation of the GABA/glutamate ratio in mutant prefrontal cortex and striatum (**g**). Graphs depict individual values, mean, and standard error (*n* = 12–14). ^*^ *p* < 0.05, ^**^ *p* < 0.01, ^***^ *p* < 0.001, statistical significance using the Mann-Whitney’s test

NF1 depletion induces region-specific alterations in GABA and glutamatergic systems. MRS was performed in the hippocampus, prefrontal cortex, and striatum of *Nf1* ^+/−^ mutant and wild-type (WT) controls (Fig. 1). We investigated whether the GABA/glutamate ratio was altered in *Nf1* ^+/−^, which is an index of the local balance between excitation and inhibition. We found a significant increase in the GABA/glutamate ratio in the mutant prefrontal cortex (*p* = 0.015) and striatum (*p* = 0.026), but not in the hippocampus (Fig. 1g). In absolute terms, *Nf1* ^*+/−*^ animals showed a small (in comparison to the effect size of local increases in GABAR, see below) but significant decrease in hippocampal GABA levels (*p* = 0.004), without significant differences in other analyzed brain regions (Fig. 1). Additionally, hippocampal (*p* = 0.006) and striatal (*p* = 0.035) glutamate levels of mutant mice were reduced (Fig. 1b, f), without changes in cortical levels (Fig. 1d).

### *Nf1*^+/−^ mutation modulates GABA(A) receptor levels in a brain region-specific manner

**Fig. 2:**
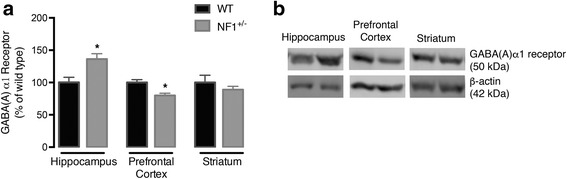
The NF1 mouse brain shows regional changes in GABA(A) α1 subunit receptor levels. Western blot analyses demonstrated that expression levels of GABA(A) receptor is up- and downregulated in hippocampal and cortical synaptosomes (**a**), respectively. Representative Western blot images of GABA(A)α1 receptor (50 kDa) and β-actin (42 kDa) are shown (**b**). Data are expressed as mean ± SEM (*n* = 8–14). ^*^ *p* < 0.05, statistical significance comparing with respectively wild-type using the Mann-Whitney’s test

**Fig. 3:**
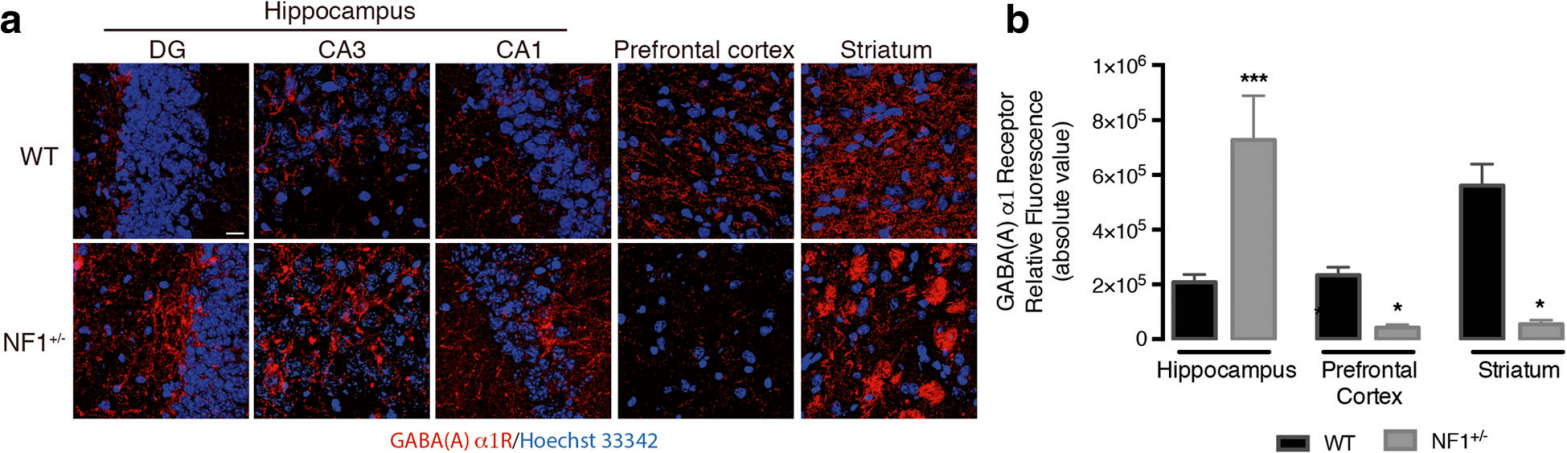
*Nf1* ^*+/−*^ mutation induced regional changes in GABA(A) α1 subunit receptor pattern. GABA(A) receptor immunoreactivity (red; Hoescht 33342—blue) was altered in *Nf1* ^*+/−*^ mice hippocampus, prefrontal cortex, and striatum, with substantial hippocampal increases, independently confirming the results shown in Fig. 2, and with reductions in the other regions, with a patchy pattern in the striatum (with both hypodense and hyperdense regions) (**a**). Accordingly, quantification of respective fluorescence intensity (**b**) demonstrated that the NF1 induced significant GABA(A) receptor augmentation in hippocampus while prefrontal cortex and striatum showed a downregulation. Data are expressed as mean ± SEM (*n* = 8–14). ^*^ *p* < 0.05, ^***^ *p* < 0.001, statistical significance comparing with respectively wild-type using the Mann-Whitney’s test. Scale bar, 20 μm

Given the demonstration that NF1-associated learning deficits are rescued by a GABA(A) antagonist [9], and our recent study in humans suggesting postsynaptic GABA changes [15], it was important to verify the impact of NF1 depletion on GABA(A) R levels. We found that NF1 is associated with a profound pattern of modification in GABA(A) R binding that seems to be region specific. Hippocampal receptor content was significantly increased in the synaptosomal compartment (*p* = 0.0270) (Fig. 2a, b). On the contrary, synaptossomes of *Nf1* ^+/−^ prefrontal cortex showed reduction in GABA(A) R α1 protein levels (*p* = 0.0012) as compared to WT (Fig. 2a, b). No changes were detected in the striatum (Fig. 2a, b). These results were corroborated by immunohistochemistry studies, as shown in Fig. 3a. Our results showed again a significant, with a quite large effect size, increase of immunoreactivity of GABA(A) R α1 in *Nf1* ^+/−^ hippocampal sub-regions, dentate gyrus (DG), and *cornu ammonis* 1 (CA1) and 3 (CA3; *p* = 0.0002), while cortical (*p* = 0.0286) brain slices exhibited immunoreactivity reduction (Fig. 3a). Furthermore, in the striatum, we observed formation of clusters surrounded by hypodense regions and reduction of GABA(A) R-expressing cells suggesting redistribution of these molecules to these hiperdense clusters (*p* = 0.0286; Fig. 3a). These results were confirmed by immunoreactivity quantification in all analyzed brain regions (Fig. 3b).

## Discussion

In this preclinical study, we found evidence for distinct pre- and postsynaptic phenotypes supporting the E/I imbalance hypothesis in ASD and related neurodevelopmental disorders but in a region-dependent manner. An important motivation was the identification of biological mechanisms in ASD and their putative heterogeneity because this is important for the design of adequate therapeutic strategies [22]. Monogenic disorders where a physiological link for impaired inhibition is present, such as NF1, are relevant models in this context [7]. Both in NF1 and idiopathic ASD, the hypothesis of E/I imbalance have been widely debated [8, 9, 13, 14, 23], and recent reviews summarize the convergence of E/I patterns in autism and schizophrenia, based on physiological and genetic evidence [1, 2]. However, in spite of the current trend to consider autism and schizophrenia as sharing molecular, synaptic, and systems targets that are common to both disorders, significant distinctions should also be considered. Moreover, although NF1 is well recognized as a single-gene model for autism [10], this notion should be put into the context of the large heterogeneity of ASD.

Here, we combined MRS, a technique previously only used in human studies, with molecular/cellular approaches, to study the hypothesis of E/I imbalance at both pre- and postsynaptic levels. We have found that the *Nf1* ^+/−^ mutation disrupted the E/I balance in a region-specific manner. Future studies should address whether this can be generalized to other ASDs and examine further whether this conceptual framework is valid across species. In particular, we discovered that the nature of E/I imbalance in hippocampus, striatum, and prefrontal cortex of mouse NF1 model presents distinct sources in spite of the presence of increased inhibitory drive in all these regions, shown in our previous work [9, 10]. These observations may be crucial to interpret the differences between the animal model and the manifestations of the human disease.

**Fig. 4:**
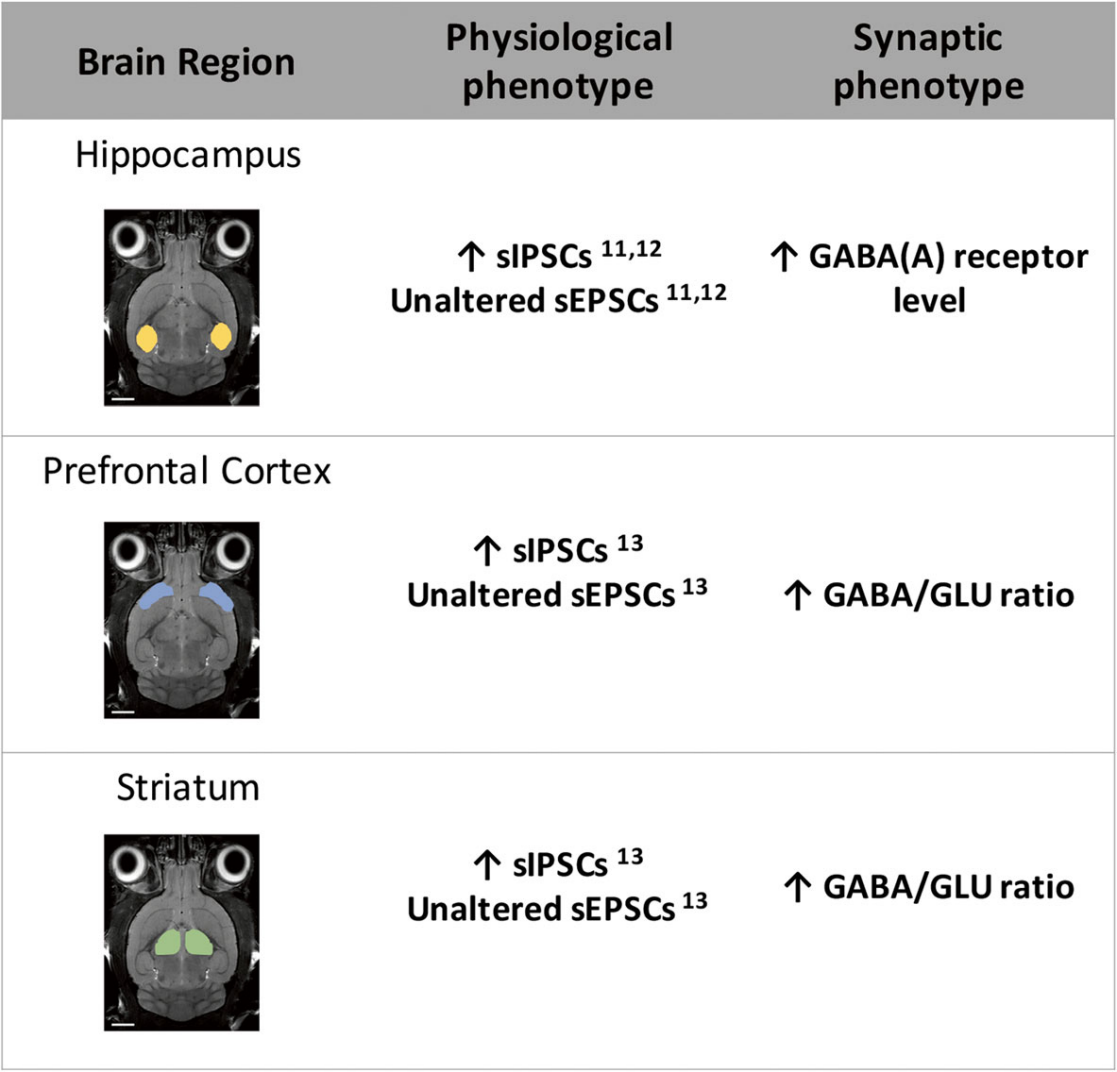
Summary of regional phenotypes of excitation/inhibition imbalance in the mouse model of neurofibromatosis type 1. The *Nf1* ^+/−^ model shows GABAergic changes in a region-dependent way. The synaptic phenotype in hippocampus is characterized by GABA(A) R disproportionate increases while in the prefrontal cortex and striatum it is characterized by GABA/glutamate ratio increases. In spite of the differences in synaptic phenotype in these brain regions, the physiological phenotype is similar with converging increases of sIPSCs relative to sEPSCs resulting in increased inhibitory drive. Abbreviation: GABA, γ-aminobutyric acid; GLU, glutamate; sEPSCs, spontaneous excitatory post-synaptic currents; sIPSCs, spontaneous inhibitory post-synaptic currents

Overall, we found that the ratio of GABA/glutamate is increased in the cortex and striatum of our animal model. The hippocampus did not show ratio changes but instead a disproportionate increase in GABA(A)R levels in spite of the small reduction of GABA levels. All these distinct cross-regional differences are consistent with increased inhibitory drive that is present in all these regions, as found in our previous work. These findings and their conceptual implications are summarized in Fig. 4.

Some care should be taken when considering absolute concentrations in the context of cross species comparisons. Although hippocampal reductions in the levels of GABA and glutamate match our observations in adults with NF1 [16], they are at odds with similar cortical alterations observed in patients but absent in mice.

Future work should establish whether the relation between regional E/I dysfunction and the pattern of deficits found in human NF1 subjects also holds true in cross-species comparisons. This was suggested to be the case in hippocampal-dependent learning impairments, visual orientation discrimination deficits, and impaired impulse control that depends on corticostriatal circuits [3, 13, 24].

In mice, deficits in executive, social, spatial memory, and attention have been associated with an increase in GABAergic neurotransmission, which dampens synaptic plasticity, in the hippocampus, pre-frontal cortex, striatum, and amygdala [8–12]. On the other hand, in NF1 patients, a decrease in GABA levels has been observed in all so far tested cortical regions [13, 14]. Here, we found that GABA and glutamate levels as well GABA(A) R expression were critically and differentially changed across regions. Differential levels of brain amino acids have been reported in several neuropsychiatric disorders [25, 26], and two recent reviews have focused on convergence and divergence of E/I phenotypes in ASD and schizophrenia [1, 2].

The inhibitory actions of central GABA are mediated in part by the ionotropic post-synaptic GABA(A)R [27]. Our data demonstrated that hippocampal levels of GABA(A) R were increased in the synaptosomal compartment suggesting increased inhibitory tone, corroborating physiological data from our previous work [9, 10]. Further, our results are similar to those observed in schizophrenic patients, showing a reduction in cortical GABA content coupled with the increased expression of GABA(A) R [28]. Despite its limitations, MRS is an important in vivo technique to address neurotransmitter changes. With this technique, it has been possible to show that global [29] and local [15] GABA levels can be related to GABA receptor concentrations.

In line with this evidence, previous neurophysiological studies that demonstrated that NF1-dependent learning deficits are caused by increased pre-synaptic GABA release that consequently disrupts long-term potentiation, with involvement of the GABA(A) R [8–12]. In the prefrontal cortex, the synaptic phenotype seems to be quite different to what was observed in the hippocampus. These results are important to understand our own previous studies which showed that NF1 affects the inhibitory system most prominently by regulating activity-dependent GABAergic release in medial prefrontal cortex [10]. Moreover, in the same study, we found that the striatum of *Nf1* ^*+/−*^ mice shows an activity-dependent increase in the frequency of inhibitory but not excitatory events. These findings are consistent with hypoactivation of corticostriatal structures that are observed in humans and directly related with working memory impairments characteristic of NF1 [10].

As a limitation, the E/I imbalance hypothesis in ASD and related neurodevelopmental disorders, although widely accepted as a valid and testable conceptual framework [1, 2], may represent an oversimplification and need refinement. Future work should directly address in vivo assessment of the dependence of excitatory and inhibitory drive on changes of the molecular phenotype at the level of the synapse.

## Conclusions

In this preclinical study, we found a relationship between tissue levels of metabolites and receptor expression in a condition where there is disruption to E/I at various levels. This provides evidence in favor of the E/I imbalance hypothesis ASD and related neurodevelopmental disorders, while showing that its nature is region-specific with distinct pre- and postsynaptic mechanisms. In the hippocampus, disproportionate expression of GABA(A) R dominates, while in the prefrontal cortex and striatum, excessive GABA/glutamate ratios are the hallmark of changes. These region-specific changes provide complementary evidence to understand the increased inhibitory tone found in our previous neurophysiological work. In spite of a similar physiological endpoint, the regional heterogeneity of molecular changes suggests the need for region tailored therapies directed to different phenotypes of autism.

## Abbreviations

ASD: Autism spectrum disorders
BCA: Bicinchoninic acid assay
CA1: *Cornus ammonium* 1
CA3: *Cornus ammonium* 3
DG: Dentate gyrus
E/I: Excitation/inhibition
ECF: Enhanced chemiofluorescence
GABA: γ-Aminobutyric acid
GABA(A)R: γ-Aminobutyric acid (A) receptor
GFAP: Glial fibrially acidic protein
IQ: Intelligence quotient
MRS: Magnetic resonance spectroscopy
NF1: Neurofibromatosis type 1
OVS: Outer volume suppression
PFA: Paraformaldehyde
PFC: Prefrontal cortex
PRESS: Point resolved spectroscopy
RARE: Rapid multi-slice acquisition refocusing echoes
SDS: Sodium dodecyl sulfate
SEM: Standard error of the mean
STR: Striatum
TE: Echo time
TR: Repetition time
WT: Wild-type
